# Supplementary figures and images for: A comparison of antigen-specific T cell responses induced by six novel tuberculosis vaccine candidates
Source: PLoS Pathog. 2019 Mar 4;15(3):e1007643. doi: 10.1371/journal.ppat.1007643 (PMC6417742; doi:10.1371/journal.ppat.1007643)

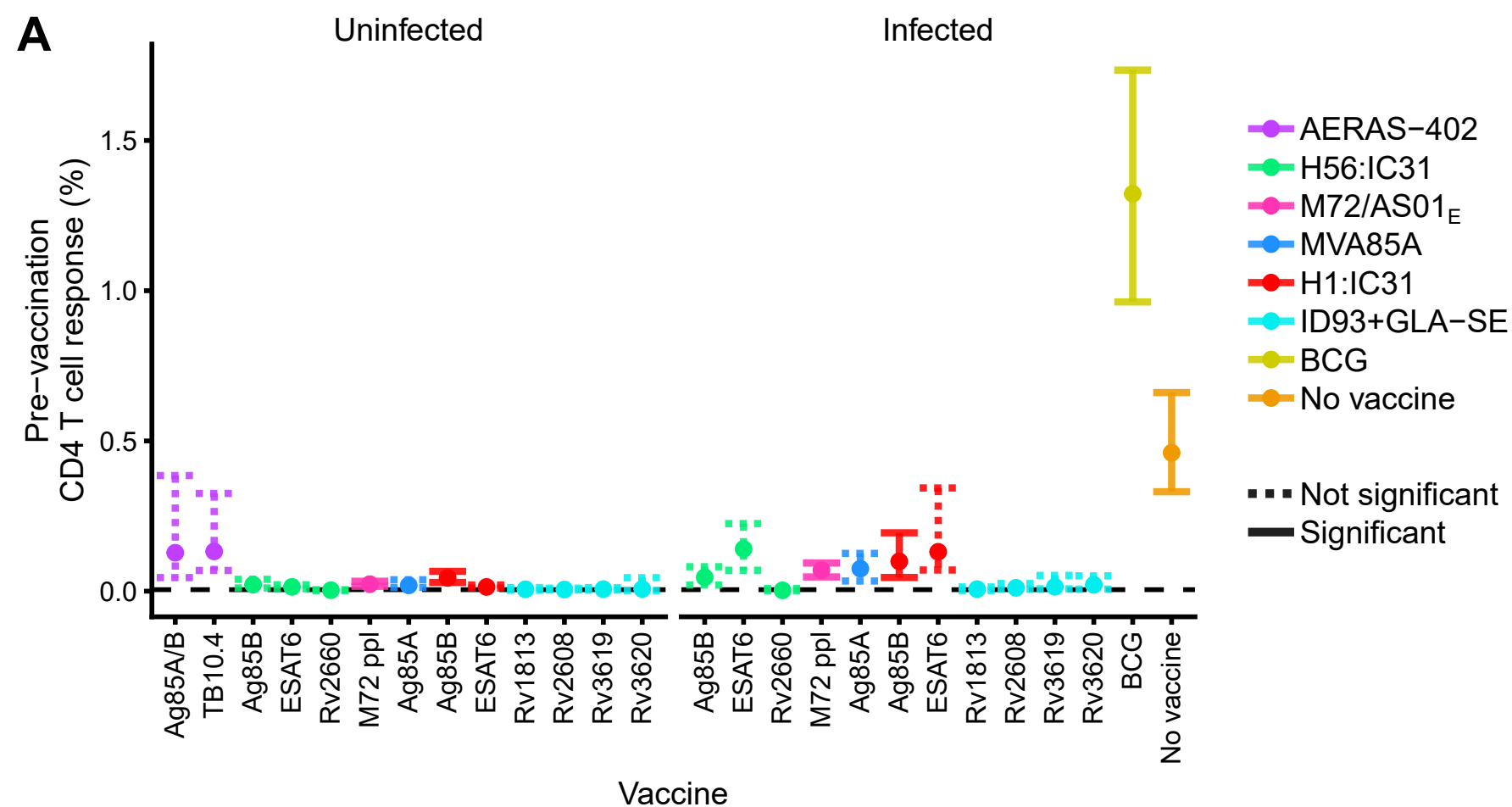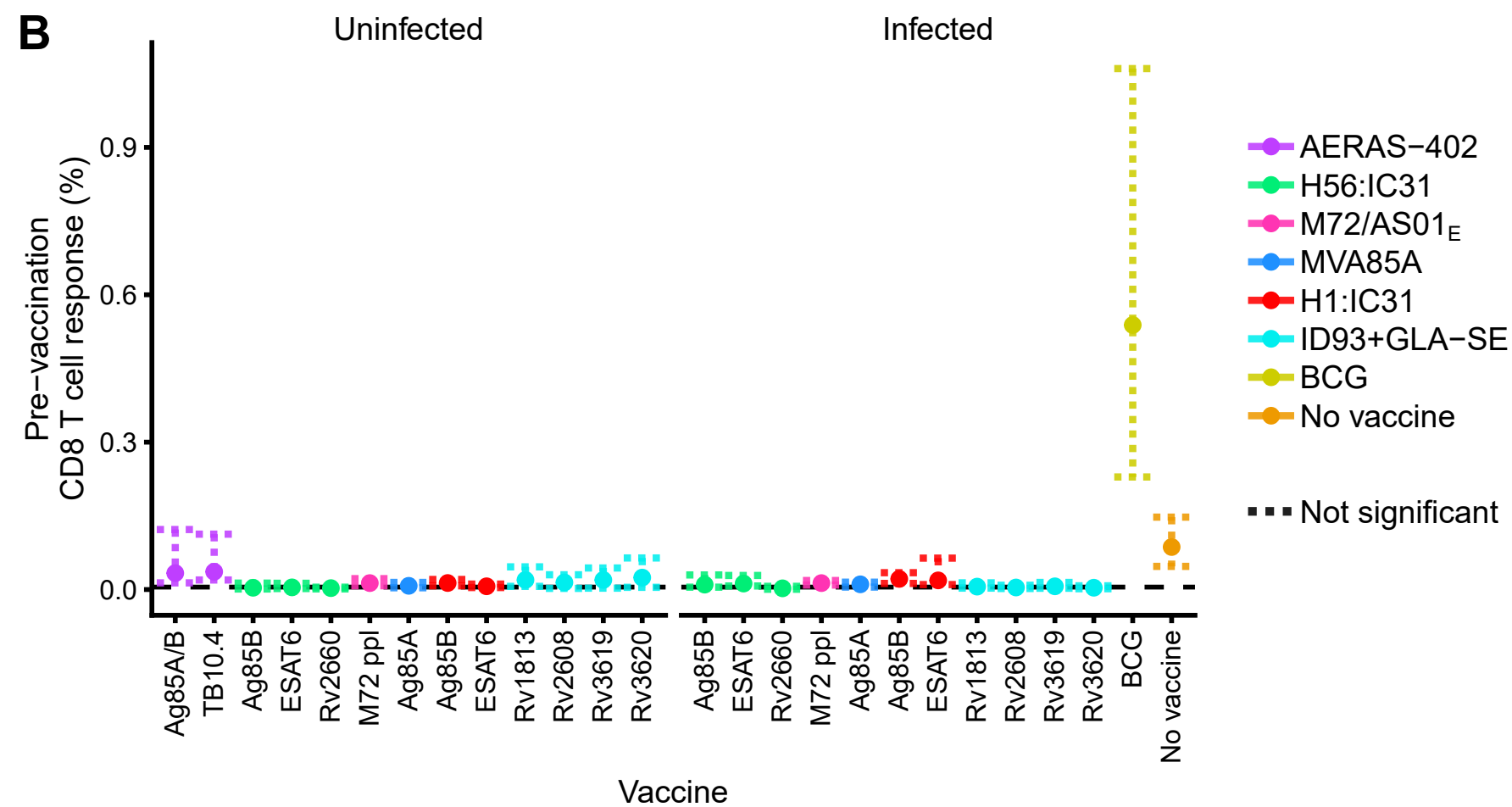

Supplement: S1 Fig — Pre-vaccination antigen-specific CD4 (A) and CD8 (B) T cell responses by individual antigens contained in each vaccine. Frequencies of antigen-specific, Th1-cytokine expressing CD4 or CD8 T cells pre-vaccination. Points denote sample trimmed means and error bars denote 95% CI. Solid error bar lines indicate responses that significantly exceeded 0.005% after controlling the false discovery rate at 0.01. Dashed lines did not meet this significance criterion. “No vaccine” indicates the immune response to M.tb infection detected after megapool stimulation in unvaccinated, IGRA-positive individuals. (PDF) [file ppat.1007643.s001.pdf]

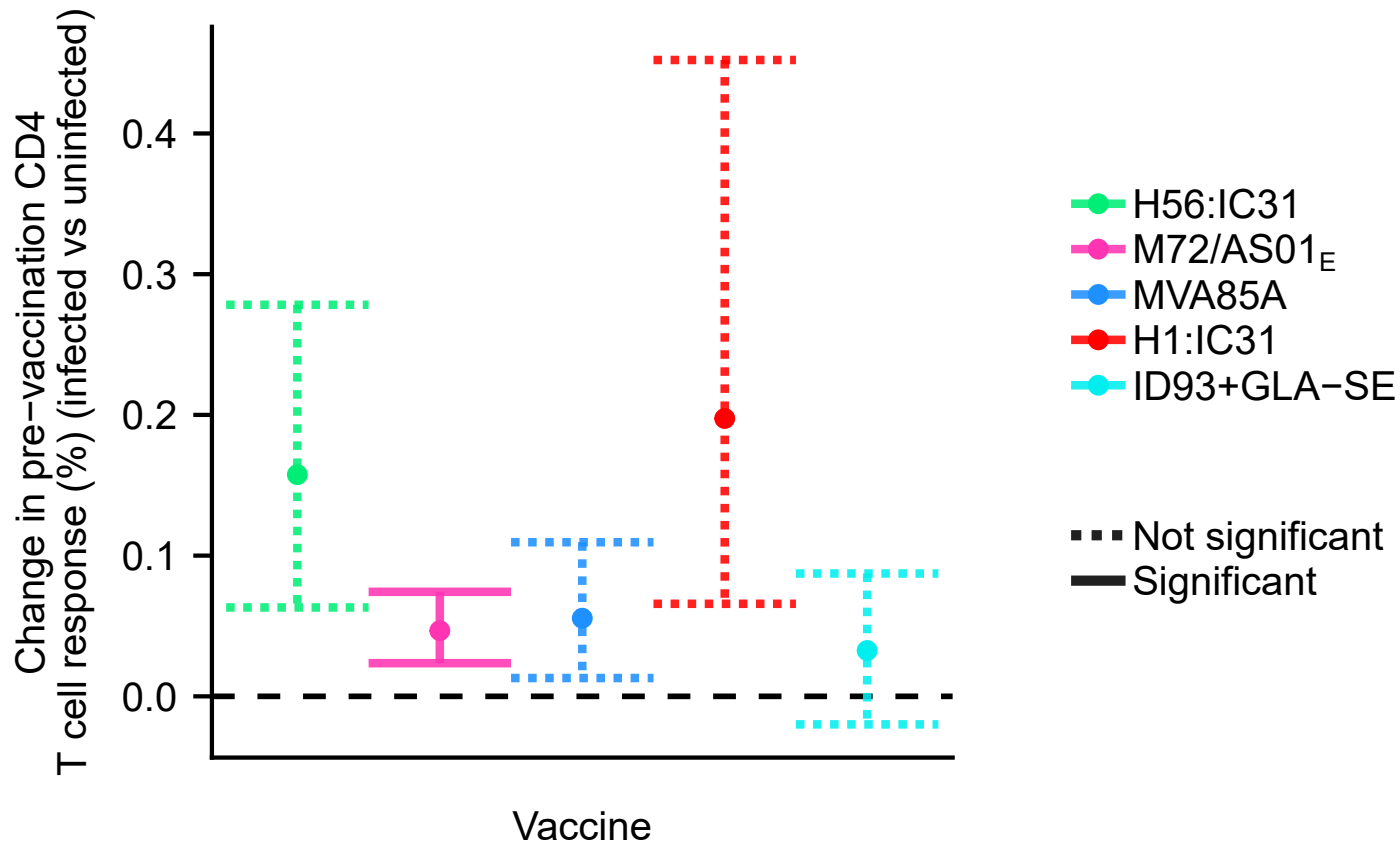

Supplement: S2 Fig — Differences between M.tb-infected and -uninfected individuals in pre-vaccination frequencies of antigen-specific CD4 T cell responses to antigens in each vaccine candidate. Points denote sample trimmed means and error bars denote 95% CI. Solid error bar lines indicate responses that were significantly different between M.tb-infected and -uninfected individuals given the same vaccine, after controlling the false discovery rate at 0.01. Dashed lines did not meet this significance criterion. (PDF) [file ppat.1007643.s002.pdf]

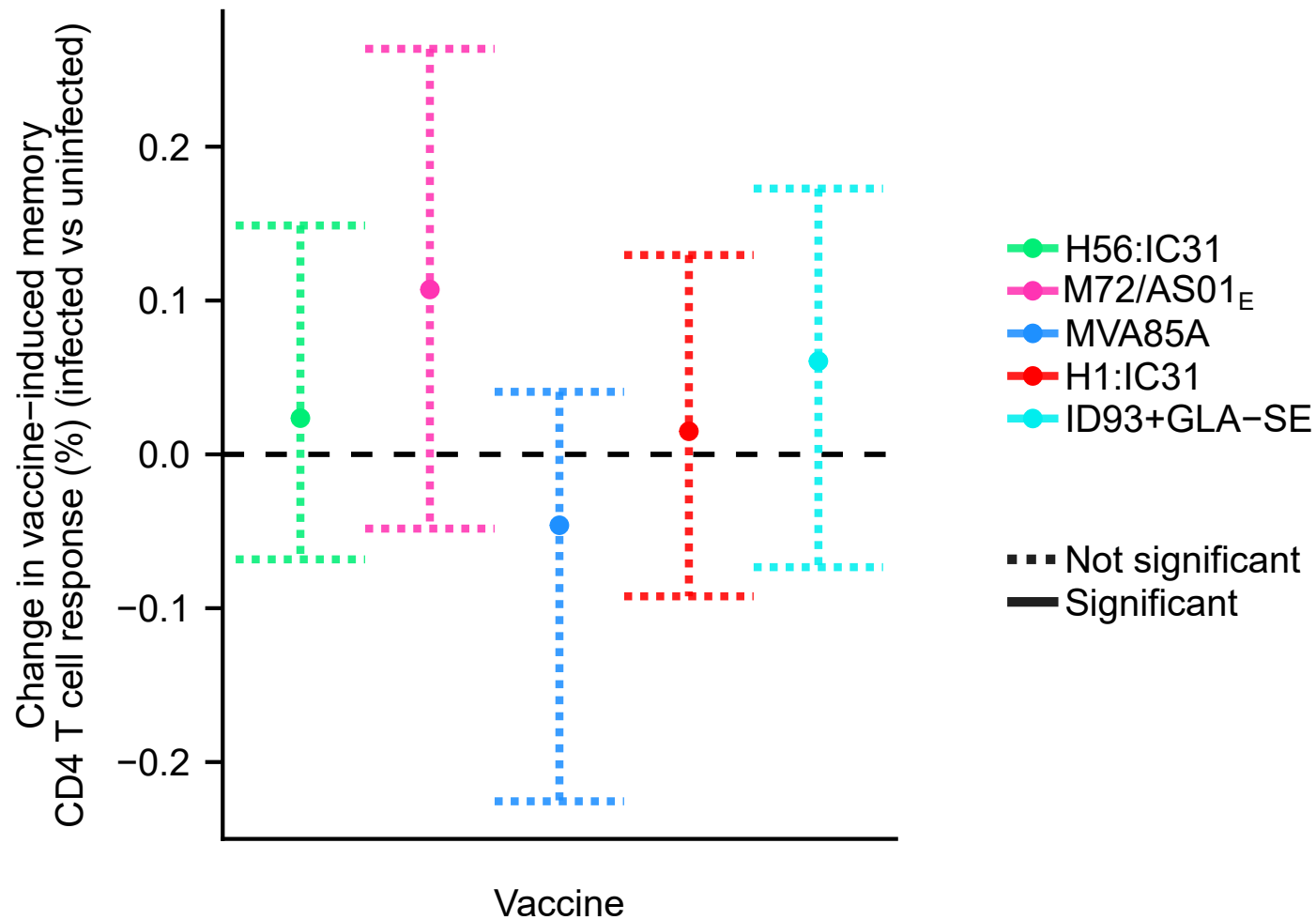

Supplement: S4 Fig — Differences between M.tb-infected and -uninfected individuals in vaccine-induced frequencies of antigen-specific memory CD4 T cell responses to antigens in each vaccine candidate (antigen-specific Th1 cytokine positive CD4 T cells at final trial time point minus pre-vaccination time point). Points denote sample trimmed means and error bars denote 95% CI. Solid error bar lines indicate responses that were significantly different between M.tb-infected and uninfected individuals given the same vaccine, after controlling the false discovery rate at 0.01. Dashed lines did not meet this significance criterion. (PDF) [file ppat.1007643.s004.pdf]

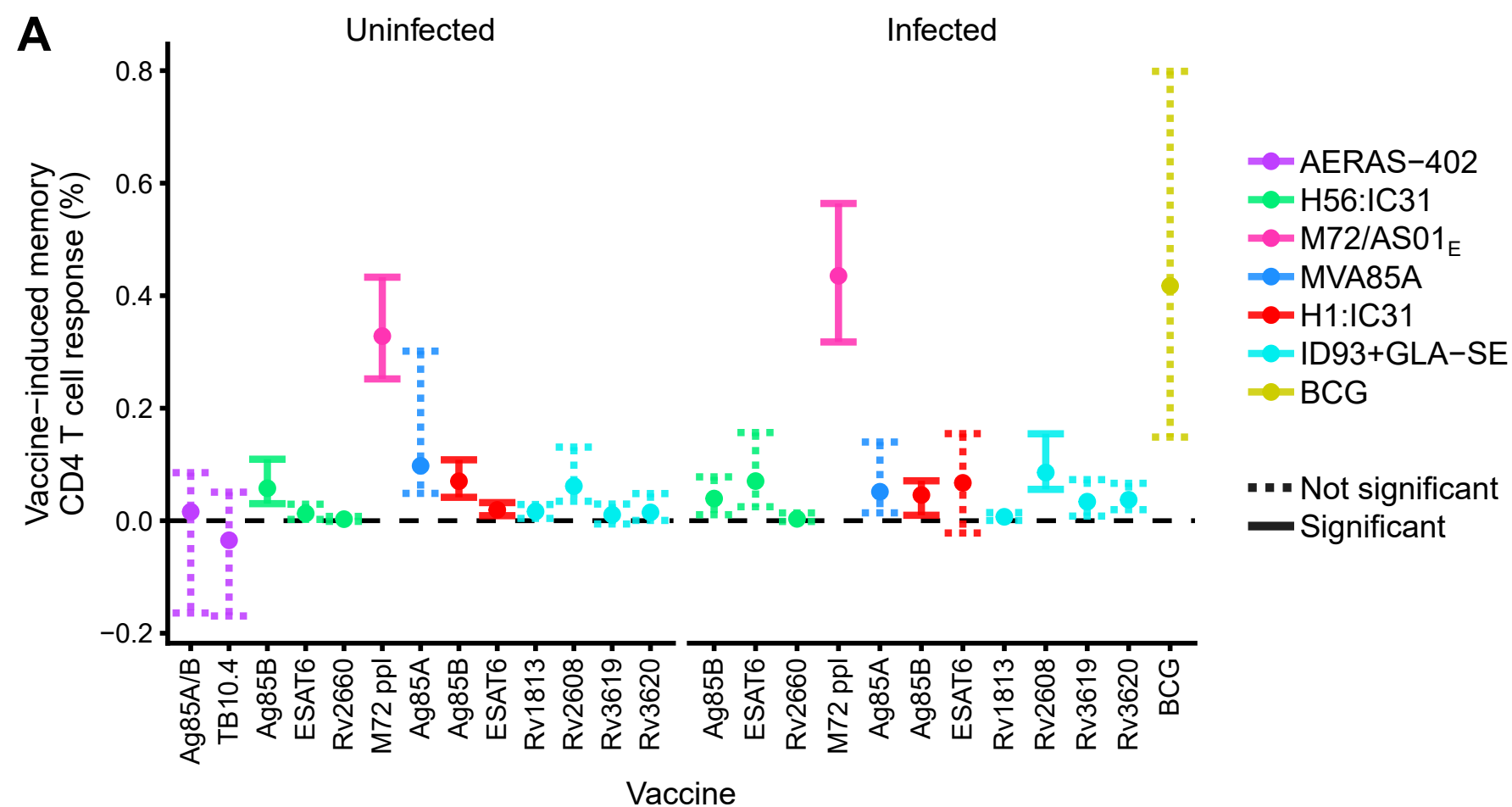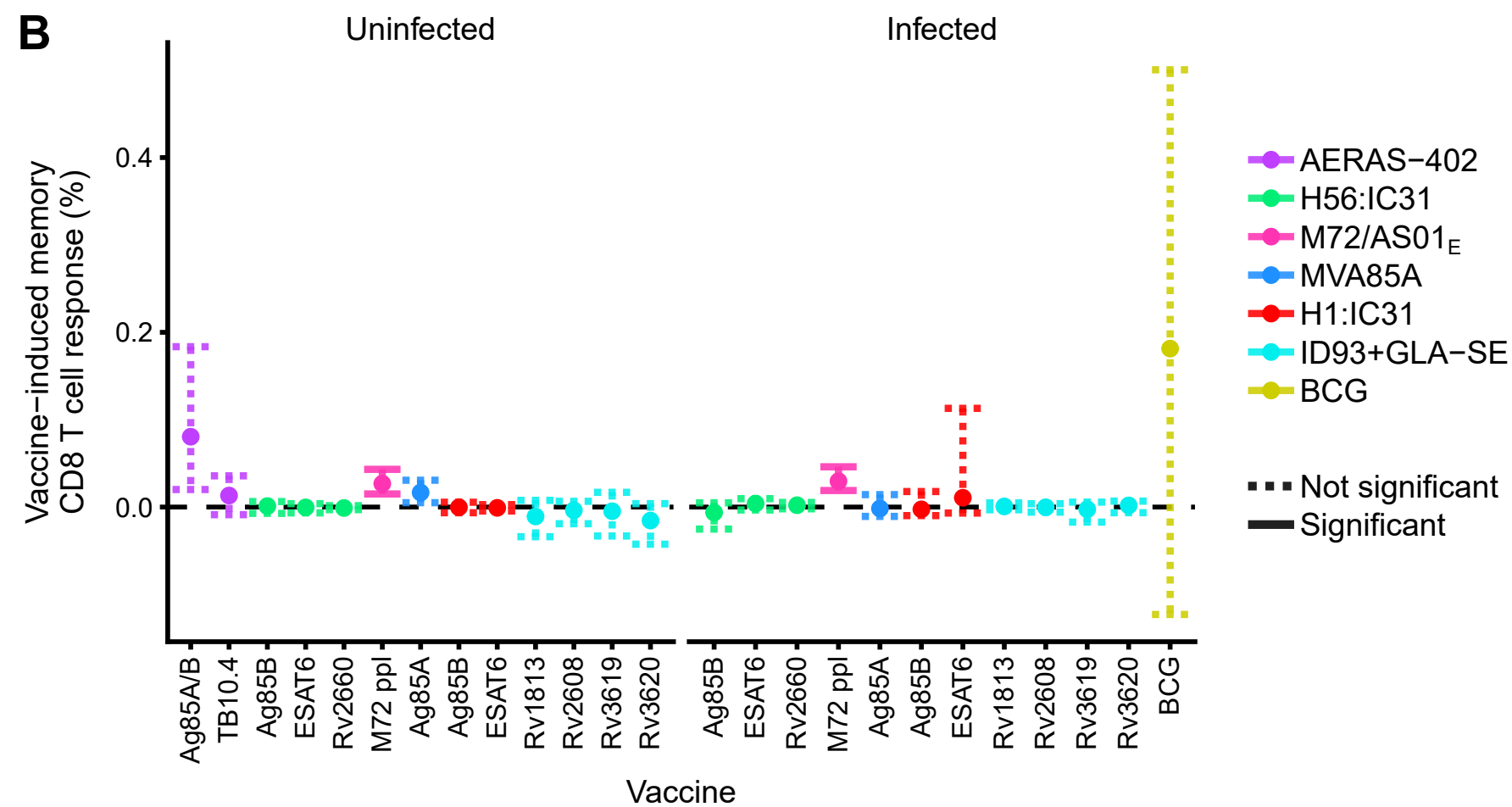

Supplement: S5 Fig — Antigen-specific memory CD4 (A) and CD8 (B) T cell responses by individual antigens contained in each vaccine. Vaccine-induced frequencies of antigen-specific memory CD4 T cell responses to antigens in each vaccine candidate (antigen-specific Th1 cytokine positive CD4 T cells at final trial time point minus pre-vaccination time point). Points denote sample trimmed means and error bars denote 95% CI. Solid error bar lines indicate responses that significantly exceeded 0.005% after controlling for a false discovery rate at 0.01. Dashed lines did not meet this significance criterion. (PDF) [file ppat.1007643.s005.pdf]

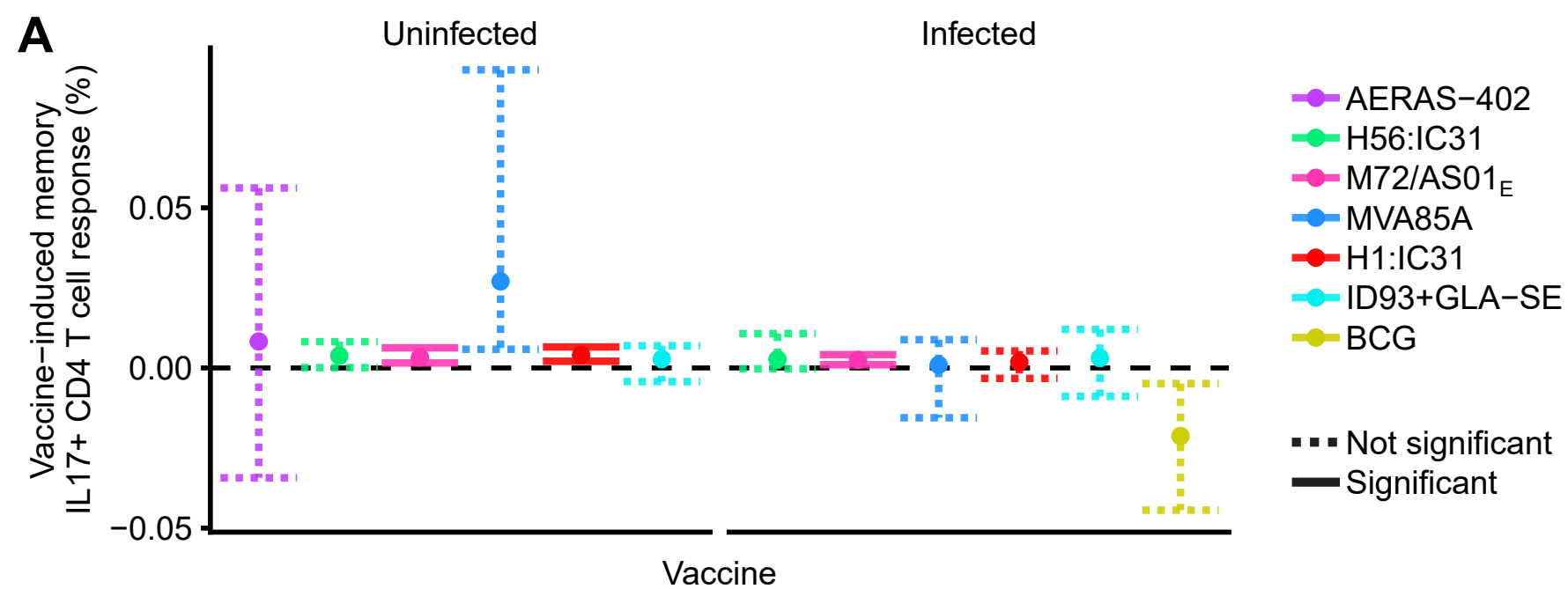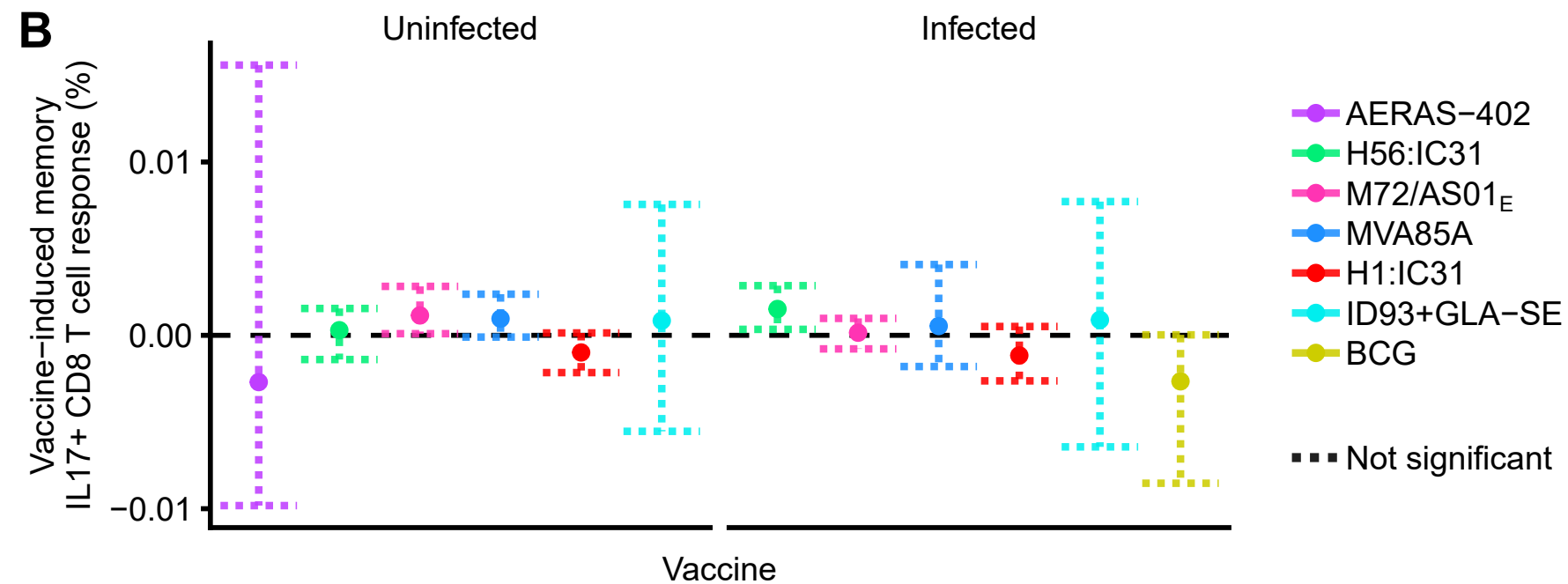

Supplement: S6 Fig — Vaccine-induced IL-17+ memory CD4 (A) and CD8 (B) T cell responses by vaccine and M.tb infection status. Frequencies of antigen-specific IL17-expressing CD4 or CD8 responses at the final time point in each trial, relative to the pre-vaccination frequencies (i.e. memory response minus pre-vaccination response). Points denote sample trimmed means, and error bars 95% CI. Solid error bar lines indicate responses that significantly exceeded 0% after controlling the false discovery rate at 0.01. Dashed lines did not meet this significance criterion. (PDF) [file ppat.1007643.s006.pdf]

**A**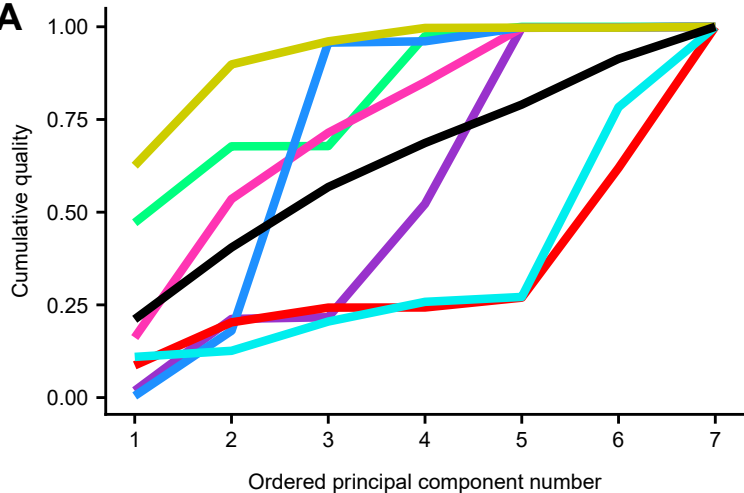

Cytokine combination

- G-2-T+
- G-2+T-
- G-2+T+
- G+2-T-
- G+2-T+
- G+2+T-
- G+2+T+
- Mean

**B**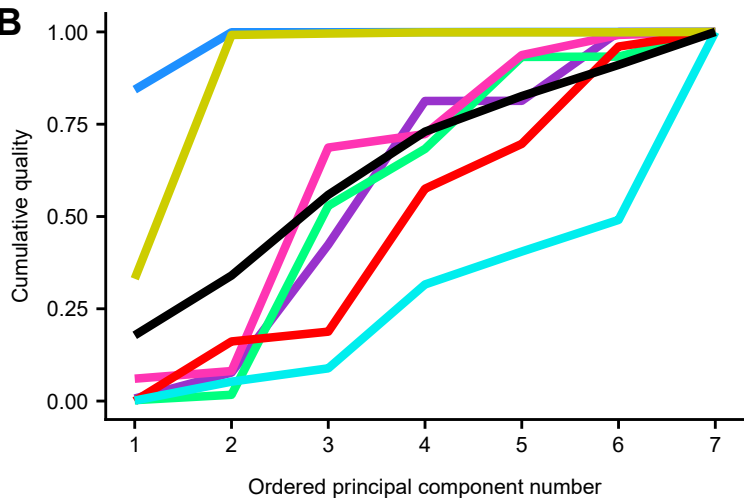

Cytokine combination

- G-2-T+
- G-2+T-
- G-2+T+
- G+2-T-
- G+2-T+
- G+2+T-
- G+2+T+
- Mean

Supplement: S7 Fig — Cumulative axis predictivity for k principal components represents the proportion of variation in the scaled vaccine-induced memory response for each cytokine combination captured by the first k principal components (see Materials and Methods for details). (PDF) [file ppat.1007643.s007.pdf]

**A**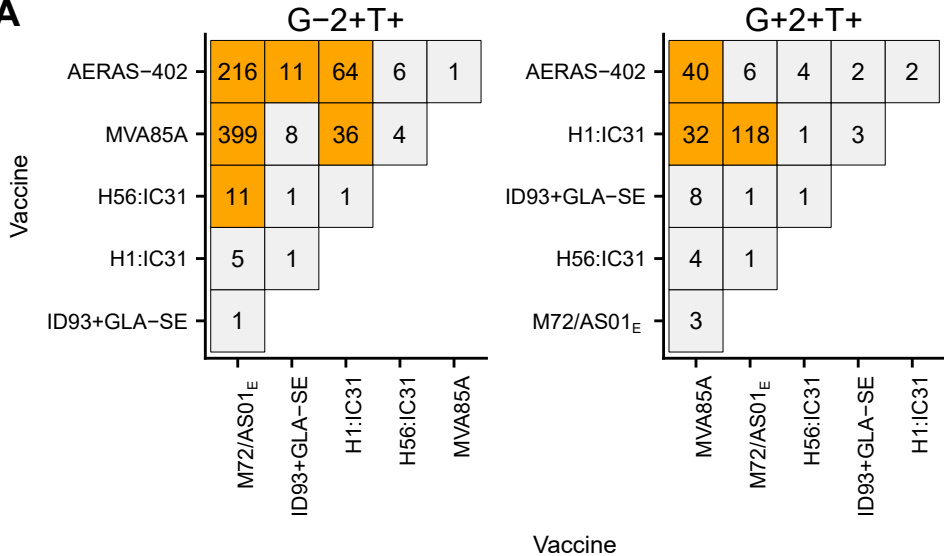**B**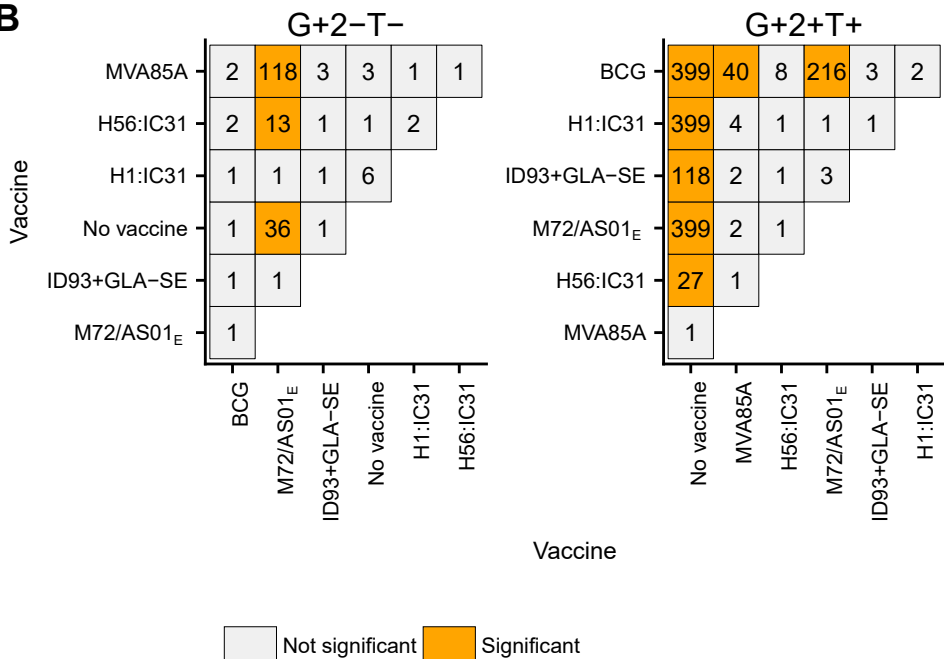

Supplement: S8 Fig — Cells display maximum Bayes factors calculated based on the p-values from hypothesis tests for a difference between vaccines in scaled vaccine-induced memory CD4 T cell responses for certain cytokine combinations in M.tb-uninfected (A) and -infected (B) individuals (see Materials and Methods for details). The colour of the blocks indicates statistical significance of the two-sided bootstrap hypothesis test for a difference in population trimmed means, after controlling the false discovery rate at 0.05. An orange block means that the induced response for the vaccine below the block was significantly larger than the vaccine left of the block. A grey block indicates non-significance. For example, the figure shows that M72/AS01E produced statistically significantly higher TNF+IL-2+ CD4 T cell responses in M.tb-uninfected individuals than H56:IC31, but not ID93+GLA-SE. (PDF) [file ppat.1007643.s008.pdf]

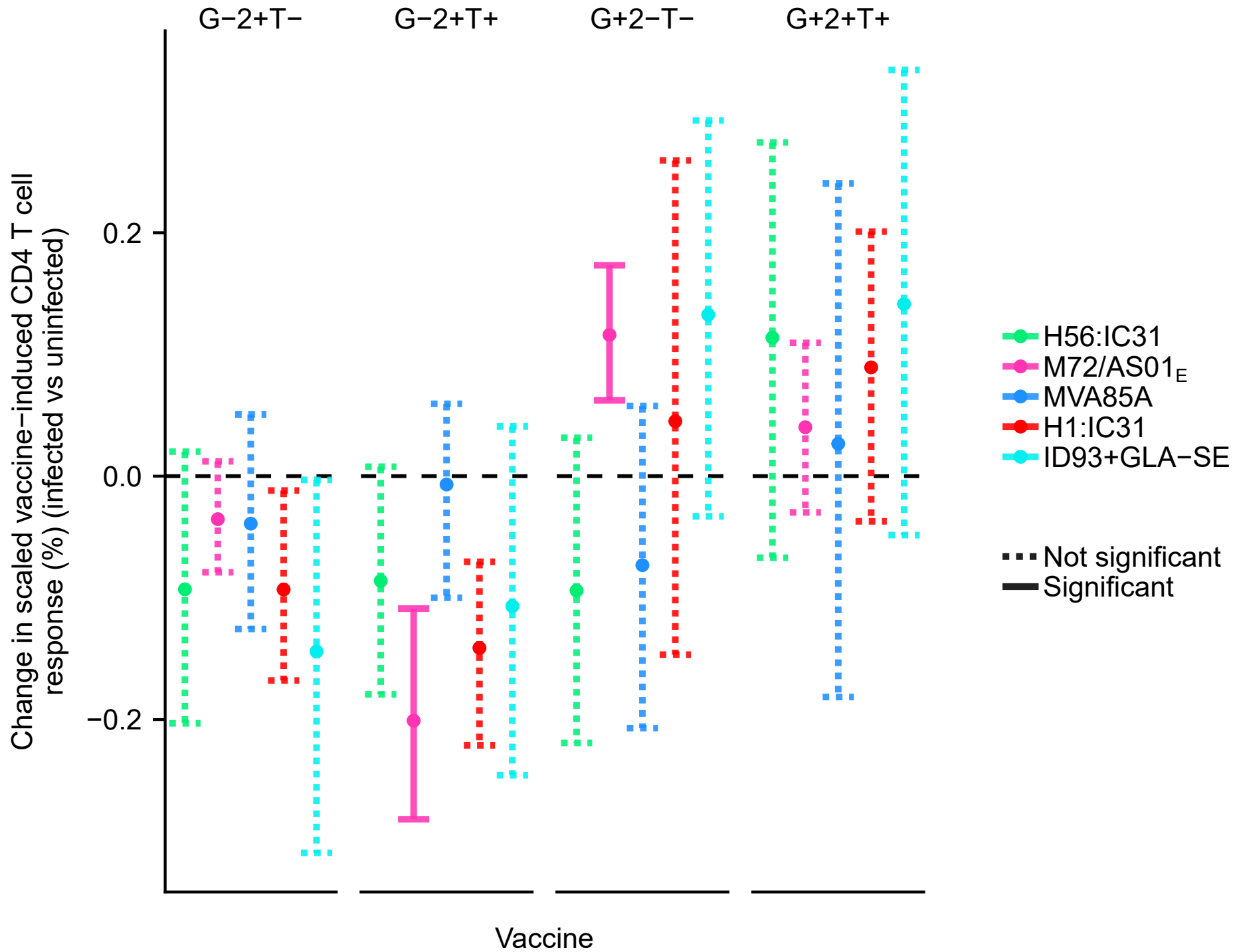

Supplement: S9 Fig — Differences between M.tb-infected and -uninfected individuals in scaled vaccine-induced frequencies of antigen-specific memory TNF+IL-2+, single IFNγ+, single IL-2+ and IFNγ+TNF+IL-2+ CD4 T cell responses to antigens in each vaccine candidate (see Materials and Methods for details). Points denote sample trimmed means and error bars denote 95% CI. Solid error bar lines indicate responses that were significantly different between M.tb-infected and -uninfected individuals given the same vaccine, after controlling the false discovery rate at 0.01. Dashed lines did not meet this significance criterion. (PDF) [file ppat.1007643.s009.pdf]
