# Supplementary material for: A comparison of antigen-specific T cell responses induced by six novel tuberculosis vaccine candidates
Source: PLoS Pathog. 2019 Mar 4;15(3):e1007643. doi: 10.1371/journal.ppat.1007643 (PMC6417742; doi:10.1371/journal.ppat.1007643)
Supplement: S3 Fig — Cells display maximum Bayes factors calculated based on the p-values from hypothesis tests for a difference between vaccines in vaccine-induced memory CD4 T cell responses (antigen-specific Th1 cytokine positive CD4 T cells at final trial time point minus pre-vaccination time point). The colour of the blocks indicates statistical significance of the two-sided hypothesis test for a difference in population trimmed means, after controlling the false discovery rate at 0.05. An orange block means that the induced response for the vaccine below the block was significantly larger than the vaccine left of the block. A grey block indicates non-significance. For example, the plot shows that in M.tb-uninfected individuals the only statistically significant differences were that M72/AS01E induced larger memory responses than all other vaccines. (PDF) [file ppat.1007643.s003.pdf]

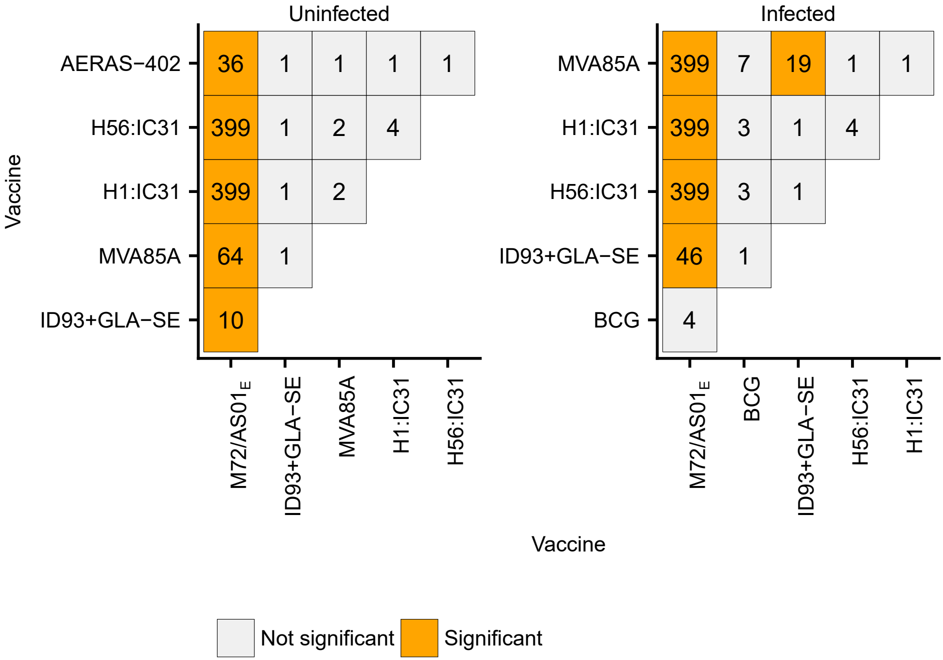

Uninfected
Infected
AERAS−402
H56:IC31
H1:IC31
MVA85A
ID93+GLA−SE
36 1 1 1 1
399 1 2 4
399 1 2
64 1
10
M72/AS01E
ID93+GLA−SE
MVA85A
H1:IC31
H56:IC31
MVA85A
H1:IC31
H56:IC31
ID93+GLA−SE
BCG
399 7 19 1 1
399 3 1 4
399 3 1
46 1
4
M72/AS01E
BCG
ID93+GLA−SE
H56:IC31
H1:IC31
Vaccine
Vaccine
Not significant
Significant
